# A Case of Naso-Pharyngeal Tumor

**Published:** 1883-07

**Authors:** F. E. Olney

**Affiliations:** Warsaw, Ind.


### Notes From Private Practice.

ARTICLE IX.

A CASE OF NASO-PHARYNGEAL TUMOR. By DR. F. E. OLNEY, Warsaw, Ind. Operation by E. Fletcher Ingals, of Chicago.

H. L., aet. 19, farmer, called upon me for treatment for catarrh, June 1, 1882.

He stated that he had been affected for about a year, having been taken with an acute coryza. After suffering for some time he had consulted a general practitioner, but he had been under treatment for the past six months and seemed to be growing worse.

Examination anteriorly revealed sub-mucous infiltration of the inferior turbinated bones, and extensive inflammation of the nasal mucous membrane.

Posterior rhinoscopy revealed a tumor of the pharyngeal vault, which subsequently proved to be attached to the basila rprocess of the occipital bone.

It so filled the post-nasal space as to render nasal respiration quite difficult, and at times impossible.

The case was treated with various sprays and insufflation, and cauterization of the enlarged turbinated bones, together with parenchymatus injections into the tumor.

After a month of this treatment, without reduction of the growth, I advised an operation.

This was acquiesced in, and July 3, 1882, we repaired to Chicago, where I had previously secured the services of Prof. E. F. Ingals.

After a careful examination, Dr. Ingals concurring in my diagnosis, the operation was performed with the Galvano-Cautery écraseur, as follows:

Having passed a strip of rubber bandage through both nasal orifices, they were brought forward through the mouth, thereby drawing the velum anteriorly, and retaining it in such a position as to enable a more thorough inspection of the parts with the rhinoscopic mirror.

The next step was to adjust the platinum écraseur, for which Dr. Ingals is entitled to much credit, since it was well calculated to tax the patience of the most skillful surgeon as well as the ingenuity of the clever mechanic.

The tumor was so large that it was impossible to carry the wire over it with the finger; however, by the aid of wire elevators, improvised by the doctor for the occasion, the wire was finally raised over the nodular surface of the tumor and secured about its base, care having been taken that the ends of the wire should not cross each other; they were next passed through a double canula electrode, which was pushed through the nostril, back to the base of the tumor. The battery was then attached, and the operation was soon completed.

There was considerable hæmorrhage, which was checked by the insufflation of tannin into the post-nasal space.

The tumor measured one and one-half inches in its antero-posterior, and one and three-fourths in its vertical diameters. Its basilar attachment was about three-fourths of an inch in diameter. It was nodulated and slightly pear shaped. No microscopic examination was made.

The result seemed to be all that could be desired, the parts healing kindly, and there was great improvement in his general health and in the condition of the nasal cavities.

Such seemed to be the condition until the end of the second month after the operation, when I found a reappearance of the tumor, which grew quite rapidly for the next two months, at which time it had gained about one-half its former size. Then it ceased to grow, and it had not enlarged when last seen by me, about six weeks ago.

The treatment since the operation has been by sprays and insufflation for the catarrh, together with an almost constant use of red clover tea.

The patient is now enjoying the best of health, and is only annoyed by the partial occlusion of the left post nasal space.

I have arranged to take the patient to Dr. Ingals for another operation as soon as he can spare the time. The doctor hopes to prevent a recurrence of the growth by repeated thorough cauterization of its stump with the galvano-cautery.

---

CONDENSED STATEMENT OF MORTALITY IN CHICAGO FOR THE MONTH OF MAY, 1883.

Total number of deaths, 910. The highest number of deaths from Phthisis Pulmonalis, 99; Diptheria, 37; Scarlet Fever, 35; Inanition, 25; Bronchitis, 43; Heart Disease, 32; Pneumonia, 60; Cerebral Meningitis, 29; Peritonitis, 11; Deaths by violence (including suicide), 50; Premature births, 24; Still births, 62; Deaths under five years, 424; under one year, 279; Males 476; Females, 433; White, 890; Colored, 20.

Comparative monthly mortality: May, 1881, 1,287; May, 1882, 1,021; April, 1883, 922; May, 1883, 910.

Highest Barometer, 30.290; Lowest Barometer, 29.484; Highest Temperature, 80.1; Lowest Temperature, 35.9.

AS a great many physicians propose to visit the forthcoming Hygienic Exhibition at Berlin, we direct attention to the advantages offered by the Berlin Policlinic, in which lectures on otology, rhinoscopy, dermatology, syphilodology, laryngoscopy, neuropathology, electrotherapy, ophthalmology, etc., are regularly delivered.

Each course commences the first of the month, and lasts thirty days.

The Berlin Policlinic is situated, Louisen strasse, 51, opposite the Charity Hospital.

